# Punctuated virus-driven succession generates dynamical alternations in CRISPR-mediated microbe-virus coevolution

**DOI:** 10.1098/rsif.2024.0195

**Published:** 2024-08-21

**Authors:** Armun Liaghat, Jiayue Yang, Rachel Whitaker, Mercedes Pascual

**Affiliations:** ^1^ Department of Ecology and Evolution, University of Chicago, Chicago, IL, USA; ^2^ Department of Biology, New York University, New York, NY, USA; ^3^ Carl R. Woese Institute for Genomic Biology, University of Illinois at Urbana-Champaign, Urbana, IL, USA; ^4^ Department of Microbiology, University of Illinois at Urbana-Champaign, Urbana, IL, USA; ^5^ Department of Environmental Studies, New York University, New York, NY, USA; ^6^ Santa Fe Institute, Santa Fe, NM, USA

**Keywords:** microbe-virus coevolution, CRISPR-Cas immunity, negative frequency-dependent selection, predator-prey, tripartite networks, dynamical transitions

## Abstract

The coevolutionary dynamics of lytic viruses and microbes with CRISPR-Cas immunity exhibit alternations between sustained host control of viral proliferation and major viral epidemics in previous computational models. These *alternating* dynamics have yet to be observed in other host–pathogen systems. Here, we address the breakdown of control and transition to large outbreaks with a stochastic eco-evolutionary model. We establish the role of host density-dependent competition in punctuated virus-driven succession and associated diversity trends that concentrate escape pathways during control phases. Using infection and escape networks, we derive the viral emergence probability whose fluctuations of increasing size and frequency characterize the approach to large outbreaks. We explore alternation probabilities as a function of non-dimensional parameters related to the probability of viral escape and host competition. Our results demonstrate how emergent feedbacks between host competition and viral diversification render the host immune structure fragile, potentiating a dynamical transition to large epidemics.

## 1. Introduction

Coevolution due to ecological interactions with natural enemies can be a major engine of intra-specific diversity, especially in the microbial world where the time scales of evolution and ecology are largely comparable. Although vast intra-specific diversity is being increasingly revealed by microbial genomics, its role in the dynamics of microbial populations and communities remains understudied. In the context of microbe–virus interactions, microbial strain-level variation can be conferred by a number of defence mechanisms used to evade viral infection. These mechanisms include mutations in receptors leading to surface resistance, innate immunity obtained with restriction-modification systems, and adaptive immunity obtained through a clustered regularly interspaced short palindromic repeats (CRISPR) system and its CRISPR-associated (Cas) proteins [1–3]. In our study, we seek to better understand the relationship between host–pathogen population dynamics and microbial strain variation induced by adaptive and heritable immune memory, as conferred by the CRISPR-Cas system [1–3]. Large epidemics where lytic viruses escape the control of microbial hosts are the central focus.

Previous theoretical and empirical studies of CRISPR-mediated virus-microbe systems have established novel coevolutionary dynamics that do not fall under the current paradigm of known modes of selection emerging from host–pathogen interactions [4–7]. These modes span fluctuating, punctuated, balancing and directional selection—as observed in (inverse) gene-for-gene and matching allele models used for plant and microbial systems (e.g. [8–12]), and in multi-strain and phylodynamic models used in human epidemiology (e.g. [13,14]). Specifically, the dynamics of CRISPR-mediated host–pathogen systems exhibit alternations between periods of sustained host control of the viral population (SHC) and sudden transitions to major viral epidemics (MVEs) accompanied by rapid host–virus co-diversification. These dynamics are transient as they, in existing models, ultimately lead to the eventual complete extinction of the viral population [4,5,15]. We refer to these transient coevolutionary dynamics hereafter as *alternating* dynamics.

The CRISPR-Cas system acts as a sequence-specific immune memory of past infections by integrating DNA fragments of invading viruses, known as ‘protospacers’ into the host’s genome as ‘spacers’ [16]. Thus, the individual phenotypes of relevance to CRISPR-mediated coevolution involve collections or repertoires of spacers and protospacers for the host and pathogen, respectively. Viral protospacer mutation and host spacer acquisition—along with viral repertoire and host memory sizes—establish a large range of possible trait combinations from which complex interaction structures can emerge. This is in contrast to the static one-to-one interactions of classic kill-the-winner models proposed for anti-viral defence mechanisms such as surface resistance and restriction-modification systems [17]. Both empirical and theoretical studies have examined the role of host strain diversity, network structure and associated metrics, particularly in the context of viral suppression. A correlation has been described between high microbial immune diversity and protection from the novel emergence of lytic viruses [2,4,15,18,19]. Quantities have been formulated to characterize immunity in terms of their distribution relative to different pathogens, which are functions of local network motifs and have been associated with the formation of host strain coalitions that persist at carrying capacity [4,15]. Pilosof *et al*. extended the understanding of the relationship between dynamics and structure by considering bipartite networks for who infects whom, and who is protected from whom. In particular, the protection or immune network was shown to develop a nested structure over time during the rapid co-diversification of major epidemics, which is associated with the initiation of the host control phase [5]. Thus, it is clear that coevolution dynamically builds complex structures towards this transition to host control.

The subsequent phase of the alternating dynamics, with the loss of microbial control and the emergence of MVEs, remains much less understood. This destabilization occurs after a series of much smaller outbreaks, suggesting a non-trivial interplay of processes that prime the availability of susceptible hosts for the transition. We know from basic models in epidemiology, with no strain variation, about the fundamental importance of a sufficient number of susceptible hosts to epidemic emergence. We address how such a critical number dynamically arises here, and the essential processes leading to the associated destabilization.

In order to address the transition to viral emergence, we replace the original hybrid deterministic-stochastic models of [4,5,15] with a fully stochastic formulation. In doing so, we explicitly account for drift associated with rare viral strains, and consequent extinctions. In particular, such viral drift is of relevance to the emergence of new strains generated by mutations. Using our stochastic model, we establish major trends in strain diversity and phylodynamics, derive the viral probability of the emergence on the basis of a tripartite network describing the structure of escape mutants, and analyse the sensitivity of the model to key, non-dimensional, ecological and evolutionary parameters. Our results establish the major role that host density-dependent competition plays in the alternating dynamics, in addition to the previously postulated adaptive and heritable immune memory. Intermittent small viral outbreaks drive stepwise decreases in host diversity rendering virus control by the host increasingly fragile. Coevolution in microbial-lytic viruses through CRISPR-induced memory is inherently non-stationary under host density-dependent competition. Possible ways to stabilize viral persistence and coexistence are discussed, as well as open areas in the context of strain variation and microbial communities.

## 2. Methods

### 2.1. Multi-type branching process model of CRISPR-mediated microbe-lytic virus coevolution

To capture the effects of drift on invasion and extinction of mutant viruses, we developed a stochastic model of CRISPR-mediated microbe and lytic virus coevolution as a multi-type branching process, implemented computationally with a Gillespie algorithm. Events are implemented as an inhomogeneous Poisson process, where time is continuous and event times are exponentially distributed with corresponding rates. Microbial hosts in our model replicate at a rate r , and viruses decay at a rate d . In addition, to model the limitation imposed by competition for common resources among the host population, we include density-dependent competition at rate rN/K , where N is the total host biomass and K denotes a carrying capacity. Here, we consider that host cellular losses (i.e. ‘washout’) occur at considerably slower rates than viral infections. Accordingly, we set the microbial washout rate to 0 such that cellular losses are primarily due to viral lysis. Furthermore, a viral strain is defined by a repertoire with a fixed number of loci g that each code for discrete trait alleles referred to as ‘protospacers’. Upon adsorption that occurs at a rate φ per particle, microbes utilize their CRISPR-Cas immune system to evade lysis with a probability q , such that one viral protospacer is randomly integrated as a ‘spacer’ into their respective genomes. The distinct collection of spacers accrued in a microbial host’s lifetime defines their immune type and their memory of previous infection. Namely, the spacers confer protection from, and cause the decay of, future viruses that carry at least one matching protospacer. Alternatively upon adsorption, with a probability of 1−q , a virus can successfully lyse a microbe and release a burst of β virion replicates. Viral protospacer mutation occurs during replication, where the probability of mutation per protospacer is μ . In our model, as in previous hybrid deterministic-stochastic formulations in [4] and [5], we impose an infinite allele assumption for viral protospacer mutation—i.e. every mutation introduces true allelic novelty to the viral population. For the description of the stochastic reactions associated with these rates and respective events, see electronic supplementary material, Model Stochastic Reactions.

**Figure 1.**
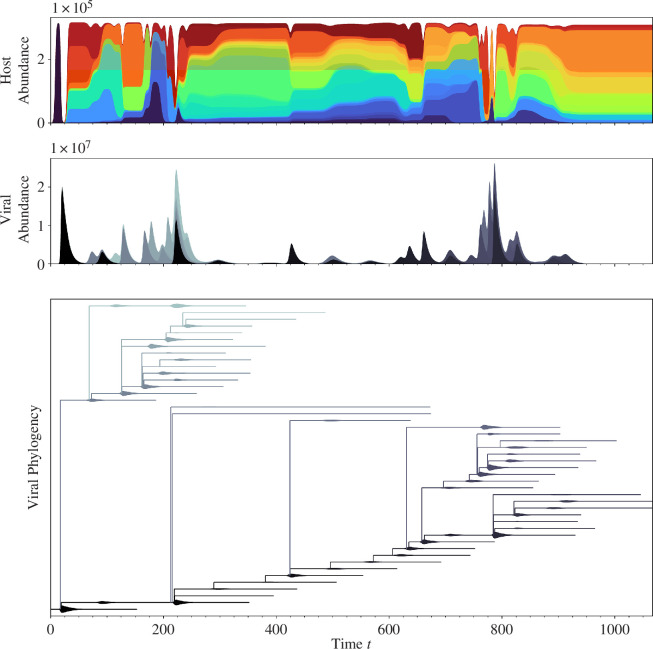
Simulated multi-type branching process model recapitulates the alternating dynamics of Pilosof *et al*. [5] and Childs *et al*. [4]. The parameter values are listed in electronic supplementary material, table S1. The plots illustrate, respectively, stacked abundances of both viral and host immune strains over time. Chromatic colours represent distinct host immune strains different by at least one spacer, and the greyscale colours represent distinct viral strains different by at least one protospacer. We designate the transient period where the host biomass is saturated at, or near, carrying capacity K as the regime of SHC, and that with large epidemics in rapid succession generated by multiple viral strains as the rapid co-diversification regime (here, near t∼200 and t∼800 ). The simultaneous diversification of the host population is more clearly observed in the forward phylogeny of figure 2*b*. Note the contrast between the viral and host phylogenies: one viral lineage dominates at a time, whereas host lineages co-occur throughout the simulation.

We find that the stochastic model recapitulates alternating dynamics in a considerable region of parameter space described later on. Figure 1 illustrates a typical stochastic simulation exhibiting these alternating dynamics. Note that both virus and microbial host populations in all our numerical realizations are initialized with 100 identical individuals and simulated until a time t=2000 . An initial oscillation of host and pathogen abundances represents the first instance of a major viral epidemic and co-diversification event, with an associated decline and rebound of the total host biomass to carrying capacity K . The rebound occurs through the growth of novel host immune strains, initiating the first period of SHC, which prevents MVEs and promotes the stochastic extinction of rare viral strains. During an SHC, sequential, small viral outbreaks occur as individual escape mutants can infect small subpopulations of hosts. These are ‘small’ in the sense that they cause relatively minor instabilities of the total host population abundance and typically target only one host genotype. This series of small viral outbreaks eventually leads to a major disturbance of host biomass that demarcates a transition to a short-lived regime of MVEs accompanied by host-virus co-diversification. Subsequently, the dynamics alternate back to another SHC period and the serial outbreaks repeat. The alternation between SHC and MVEs is terminated through eventual extinction of the virus population. For a given parameter set, the stochastic nature of the system is reflected in the varying dynamics in different realizations, e.g. the number of SHCs for parameters used in figure 1 can vary typically from zero to three. Here, we slightly increased values of spacer acquisition probability q and protospacer mutation probability μ , used by Pilosof *et al*. [5], to counteract the effect of drift on the emergence of novelty. Among 50 realizations, this increase allows us for the probable emergence of a number of alternations between the SHC and MVE regimes before viral extinction.

**Figure 2.**
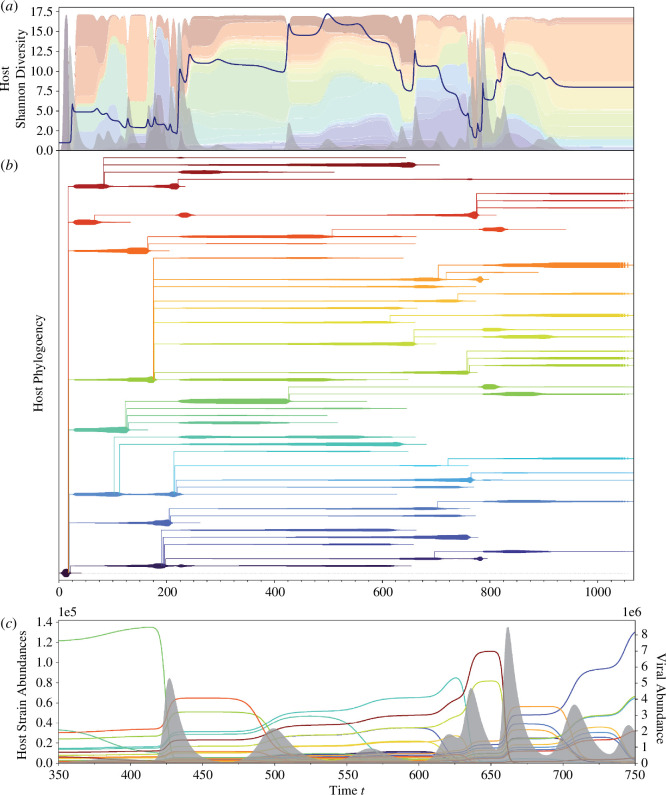
Virus-driven host succession underlies dynamical transition to MVEs (*a*) Shannon diversity of host immune strains over time represented by the dark blue curve. The multi-coloured stacked abundances and filled-in grey abundances represent the host immune strains and total viral populations, respectively. Each colour corresponds to a branch in the forward phylogeny of (*b*), and a curve in (*c*). We observe that, during each transient period of SHC, host Shannon diversity exhibits a decreasing trend. Transitions to the MVE regime are strongly associated with critically low levels of host Shannon diversity, after which a new transient period of SHC initiates. (*b*) Forward phylogeny of host immune strains. Each colour in the phylogeny represents a distinct host immune strain, corresponding to the colours in (*a*) and (*c*). The closer the hue of two strains are, the more likely they belong to the same clade. Note that close hues do not necessarily signify a similar immune profile. The width of each branch represents the absolute abundance of each strain. The collapse of immune strains are associated here to diversification events that occur due to viral epidemics, as immune strains acquire one additional spacer and thus increase the size of their respective arrays. The transitions to MVEs and associated rapid co-diversification occur at temporal windows near t∼200 and t∼800 . Electronic supplementary material, figure S2 shows that richness and the size of the spacer array exhibit an increasing trend over time. (*c*) A zoomed-in depiction of host immune strain collapse upon a viral epidemic. Here, coloured curves represent host immune strain absolute abundances corresponding to colours in (*a*) and (*b*), and the filled-in grey curve represents absolute viral abundance. As epidemics grow in size, the population of at least one host immune strain correspondingly declines.

## 3. Results

### 3.1. Virus-driven succession of host immune strains, mediated by host density-dependent competition, potentiates viral escape and associated major epidemics

The observations of the stochastic realization depicted in figure 1 suggest a series of events that build towards the destabilization of host control. We describe in this section a series of characteristic trends in the temporal dynamics, which shed light onto key processes influencing diversity of both host and pathogen.

During a period of SHC, a set of co-occurring host immune strains partition the overall carrying capacity and undergo serial reorganizations of dominance induced by small viral outbreaks usually targeting a single strain (i.e. disturbances). This is a process of succession that characteristically demonstrates non-stationary diversity dynamics (figure 2*a*,*b* and electronic supplementary material, figure S1). Notably, host strain Shannon diversity exhibits an overall decreasing trend, where SHC-to-MVE transitions are associated with its low levels (figure 2*a*). Each viral outbreak during SHC corresponds to the collapse of at least one dominant immune strain (figure 2), and thus leads to the increase in abundance of co-occurring immune strains. In some instances, these outbreaks cause small localized increases of Shannon diversity. As expected, the forward phylogeny in figure 2*b* reveals that declines of dominant immune strains are accompanied by the acquisition of novel spacers, consequently generating novel immune strains introduced at rare abundances.

Host density-dependent competition generates the conditions for punctuated succession. Following the collapse of at least one dominant immune strain upon a small outbreak, co-occurring immune strains increase in abundance as the strength of host density-dependent competition weakens (as a linear function of host biomass N : rN/K ). This implicitly represents the growth of co-occurring host strains due to newly available resources. Namely, cohorts of more abundant immune strains are able to increase in abundance as they clonally expand, whereas more recently generated cohorts with newly added spacers accumulate but remain rare. This is due to established cohorts typically exhibiting higher abundances, which allows for more rapid growth than that of more recent cohorts (a consequence of exponential growth). Electronic supplementary material, figure S2 demonstrates that although newer strains accumulate during SHC, the overall population is represented mostly by older strains with similar times of creation. This reorganization of immune strains allows for an overall decreasing trend in Shannon diversity, despite possible local increases in richness due to spacer acquisition (electronic supplementary material, figure S1). As the total host population rebounds, host density-dependent competition maximally strengthens and the population transiently returns to carrying capacity. This gives rise to the observed punctuated behaviour of succession. Note that in a scenario of unbounded growth, all co-occurring immune strains would exponentially grow and evenness of frequencies would also increase. Thus, host density-dependent competition, in conjunction with serial viral outbreaks, is crucial for the decreasing trend of diversity to emerge. Following the end of an SHC, Shannon diversity rapidly rises as the final dominant immune strain(s) collapse. As the subsequent SHC establishes, the expected time of creation of the rapidly rising strains markedly increases (electronic supplementary material, figure S2). In other words, each period of SHC is largely represented by immune strains that are considerably ‘newer’ than those of preceding periods. On average, these strains have a greater number of spacers than those of their ancestors in the preceding period of SHC (electronic supplementary material, figure S1).

**Figure 3.**
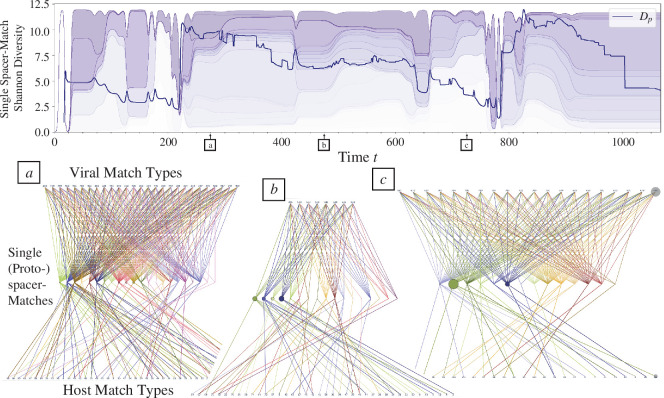
Tripartite escape networks reveal that during periods of SHC the majority of the host biomass progressively becomes associated with fewer single-spacer matches. This is shown by the Shannon diversity $D_{p}(t)$ of single-spacer matches, weighted by host immune strain frequencies (in the dark blue curve). This quantity, directly computed from the tripartite escape network, captures the diversity of single matches protecting access to the dominant immune strains of the host population (see (3.1) for reference). $D_{p}(t)$ exhibits a decreasing trend during a host control period. (*a*–*c*) Diagrams illustrating the tripartite escape network at times 275, 475 and 750, respectively. The viral and host nodes depict match types that represent a unique set of single (proto)spacer matches. Therefore, each node can represent multiple different virus/host strains as long as all have the same set of single (proto)spacer matches to the host/virus population. The size of the viral and host nodes represents the abundances of the respective match types. The node size of the single-spacer matches in the centre layers represents the sum of host abundances connected to the respective spacer.

In order for the host Shannon diversity to be driven to its low levels each viral outbreak must successfully generate an escape variant for a subsequent outbreak to occur. Namely, as susceptible hosts are often entirely lysed upon an outbreak (figure 2b and electronic supplementary material, figure S3), viral strains must escape the immune protection of co-occurring host competitors. Otherwise, viral decay following ‘burnout’ can cause the extinction of a lineage. The probability of at least one escape variant is a function of the number of susceptible hosts infected (this relationship is later described mathematically in (3.3). As the maximum achievable size of the susceptible biomass is limited by the carrying capacity established by host density-dependent competition, so is the maximum probability of at least one escape variant.

**Figure 4.**
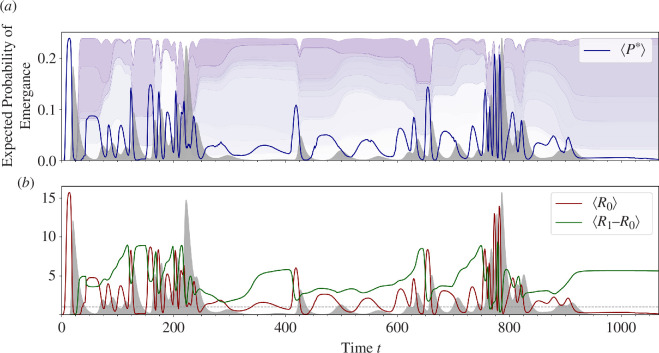
(*a*) Represented by the dark blue curve is the expected probability of emergence over time $\left\langle P^{*}\right\rangle$ , weighted by infection probabilities. Its variation is indicative of small outbreaks and of the transition to large ones. Namely, the local maxima of $\left\langle P^{*}\right\rangle$ directly correspond to the onset of viral epidemics that are represented by the filled-in grey curves. As the time series approaches a transition to rapid co-diversification, $\left\langle P^{*}\right\rangle$ begins rapidly cycling, serving as an early warning signal of this change in the dynamics. (*b*) Decomposition of $\left\langle P^{*}\right\rangle$ over time: plots of expected reproductive ratio $\left\langle R_{0}\right\rangle$ and expected reproductive escape differential of rank 1, $\left\langle R_{1}-R_{0}\right\rangle$ . The dashed grey line represents $\langle R_{0}\rangle =1$ . When the reproductive escape differential reaches a local maximum, it is then directly followed by a local maximum of $\left\langle R_{0}\right\rangle$ . This is particularly evident in the temporal interval of [575,675] . See electronic supplementary material, figure S6 for similar correspondences observed for reproductive escape differentials of lower ranks: $\left\langle R_{2}-R_{0}\right\rangle$ , $\left\langle R_{3}-R_{0}\right\rangle$ .

To examine associated patterns of escape, we consider the configuration of virus-spacer-host matches in a tripartite network. This tripartite escape network indicates the single (proto)spacer matches which a given viral strain needs to mutate to escape and infect the specific host strains solely protected by this single match (see example networks in figure 3 and electronic supplementary material, figure S4*a*). Note that due to the low protospacer mutation probability μ , escape from host strains that exhibit protection via a double spacer match or higher is negligible. In figure 3, we compute the Shannon diversity of the single-spacer matches of the tripartite network over time, weighted by immune strain frequencies, expressed as


(3.1) $$\begin{array}{r} D_{p}(t)=\prod_{\sigma \in S}e^{-\frac{N_{\sigma }}{N_{\text{tri}}}\ln\frac{N_{\sigma }}{N_{\text{tri}}}} \end{array}$$

where S is the set of spacers that confer a single match between hosts and viruses in the tripartite escape network. $N_{\sigma }$ is the total biomass of immune strains singly matched with the viral population with a spacer σ . $N_{\text{tri}}$ is the total sum of host biomass represented by the tripartite escape network, normalized for host strains that are represented by multiple spacers. This quantity, directly computed from the tripartite escape network, captures the diversity of single matches protecting the dominant host strains from infection, and therefore, the potential escape pathways for the virus to access protected host biomass. We find that $D_{p}(t)$ exhibits a decreasing trend during SHC, with low values corresponding to the transition to the MVEs regime. This pattern, together with the concomitant decreasing trend in Shannon diversity of the dominant host strains, reflects a concentration of host biomass in a few escape pathways such that host control is rendered fragile. Through the successive reorganizations of host biomass, a low number of escapes is poised to provide access to a majority of the host biomass. The tripartite escape networks depicted in figure 3 sampled at three different times illustrate the progressive concentration of host biomass leading to fragile control. Initially, spacer node sizes are representative of an approximately uniform distribution of host abundances, and as host strains collapse due to small viral outbreaks, a few spacer nodes grow to represent a considerably higher proportion of host abundance.

The decreasing trend in escape pathways can be visualized in more detail by overlaying, on the viral phylogeny, the Shannon diversity of single matches computed for each viral strain (electronic supplementary material, figure S4*c*). Due to past epidemic burnouts, successful outbreaks are those generated by viral strains that are escape variants of their mother strain—each escape priming the viral population for another. Through the forward phylogeny, descendant strains exhibit a lower diversity of single matches than their parent (electronic supplementary material, figure S4*d*)

### 3.2. Probability of viral evolutionary emergence predicts the onset of small viral outbreaks and cycles more frequently in the approach to major epidemics

Because of the stochastic nature of the dynamics, it is of interest to derive a quantity that captures the effects of drift on the emergence of rare viral variants for our multi-type branching process model. The probability of viral evolutionary emergence is of particular interest, as it captures the ultimate fate of both an individual viral strain introduced at rarity and its daughter mutants (i.e. the lineage established by an individual viral strain). Here, we formulate the probability of emergence given an arbitrary immune composition of the host population. To this end, we rely on the bipartite infection network (who infects whom) and the tripartite escape network, to obtain an expression that applies to any structure of such networks. This is in contrast to a previous study by Chabas *et al*. [19], wherein the probability of viral emergence is computed for a composition of immune strains with equal proportions and single-spacer matches. In addition, our computation of probability of viral emergence includes a viral burst size β as implemented in our branching process model.

We first define a viral match phenotype i to be the collection of protospacers of a virus that exists in the entire host pool of immunity. We refer to this alternative phenotype as a viral match phenotype (see electronic supplementary material, Probability of Viral Evolutionary Emergence for further explanation). We derive an expression for the probability of emergence of a virion sampled by immune strain densities at a time t (electronic supplementary material, equation S12). In our model, viral lysis is the driver of host mortality, and any substantial decline of the host population implies the emergence of an associated viral epidemic. Hence, we consider the host population effectively constant during the periods that lead up to an epidemic of a viral match phenotype i . In doing so, the resulting solution of electronic supplementary material, equation S12 captures the early stages of an epidemic, before the host population is considerably lysed. For a viral match phenotype i , the probability of emergence expanded around a small protospacer mutation probability has the following general form:


(3.2) $$P_{i}^{*}=\underset{\text{infection network contribution}}{\underbrace{\frac{R_{0i}}{R_{0i}+\beta }+\frac{W_{0}(z_{0i})}{\beta }}}+\underset{\text{tripartite network contribution}}{\underbrace{\mu \;F(R_{0i},R_{1i},\ldots ,R_{\ell i})+O(\mu^{2})}}.$$

The derivation of solution (3.2) is detailed in electronic supplementary material, Probability of Viral Evolutionary Emergence. A viral match phenotype i can mutate into a single-match escape j . The single match escapes j are indexed by the integer set [1,ℓ] where each element of the set indicates the rank of the accessible host biomass; i.e. j=1 is the phenotype that provides access to the largest host biomass whereas j=ℓ provides access to the smallest. Accordingly, $R_{0i}$ represents the reproductive ratio of a viral match phenotype i at time t (where t is omitted), and $R_{ji}$ represents the reproductive ratio of a viral match phenotype i upon a single-match escape to phenotype j at time t (more technically, $W_{0}(z_{ji})$ is the principal value of the Lambert function of $z_{ji}$ , and $z_{ji}=-\beta R_{ji}e^{-\frac{\beta R_{ji}}{R_{ji}+\beta }}/(R_{ji}+\beta )$ ). For equations of the form $z=xe^{x}$ , Lambert functions are solutions: $x=W_{k}(z)$ where k is an integer representing branches in the complex plane). In solution (3.2), we observe that the different components of the emergence probability are expressions of different reproductive ratios (defined as is typical in epidemiology, as the number of daughter virions generated within the lifetime of a viral strain). The first two terms in expression (3.2) originate from the bipartite infection network and reflect the availability of current susceptible hosts for a viral strain. The following function F and the factors associated with higher order protospacer mutation probability μ originate from the tripartite escape network, reflecting growth upon hosts that become available upon escapes (see electronic supplementary material, Probability of Viral Evolutionary Emergence for complete form of function F ).

We consider expression (3.2) as an expectation $\left\langle P^{*}\right\rangle$ weighted by the normalized infection probabilities of the viral match phenotypes at a time t . Thus, the expectation $\left\langle P^{*}\right\rangle$ represents the expected probability of emergence of an individual virion sampled by the host population for infection at a time t (figure 4*a*). The local maxima of $\left\langle P^{*}\right\rangle$ successfully corresponds to the onset of every viral outbreak. As we would expect, a comparison with electronic supplementary material, figure S4c reveals that instances where $\left\langle P^{*}\right\rangle$ rises towards a maximum correspond to the recent generation of an escape variant that is at rare abundance. More frequent cycling of $\left\langle P^{*}\right\rangle$ is also observed as MVEs approach, indicating proximity to the SHC-to-MVEs transition. We can also depict the contribution of specific reproductive ratios to $\left\langle P^{*}\right\rangle$ , via $\left\langle R_{0}\right\rangle$ and $\left\langle R_{1}-R_{0}\right\rangle$ (figure 4*b*). We refer to $\left\langle R_{1}-R_{0}\right\rangle$ as the *reproductive escape differential of rank 1*, as it represents the number of *additional* daughter virions that can be generated within the lifetime of a viral strain, upon a single escape from the largest (first ranked) host biomass. The local maxima of $\left\langle R_{1}-R_{0}\right\rangle$ directly precede, and correspond in size to, the maxima of $\left\langle R_{0}\right\rangle$ . Similar correspondences of optima can be observed for lowers ranks: $\left\langle R_{2}-R_{0}\right\rangle$ and $\left\langle R_{3}-R_{0}\right\rangle$ (electronic supplementary material, figure S6). These correspondences between local maxima reflect that viral outbreaks during SHC periods are characteristically preceded by transitions of microbial biomass from protected to susceptible hosts (more technically, from the tripartite escape network to the bipartite infection one), which provide a pool of susceptible hosts for a viral outbreak. We note that $R_{mut}$ from [5] captures the expected reproductive ratio of a new viral mutant in a deterministic context, whereas $\left\langle P^{*}\right\rangle$ captures the potential of a lineage, established by an extant viral strain, to stochastically generate an outbreak locally in time.

### 3.3. Alternations become more likely with increases in the maximal adsorption ratio, viral burst size and protospacer mutation probability

Given the established role of viral escape in SHC-to-MVE transitions, we derive the maximum probability of a viral strain generating at least one single-match escape variant, and explore the sensitivity of the number and duration of SHC periods to changes in parameters implicit to the probability.

The probability of a viral strain generating at least one single-match escape upon contact with S susceptible hosts is $P_{S}(n_{e}\geq 1)=1-(q+(1-q)(1-\mu {)}^{\beta }{)}^{S}$ , where $n_{e}$ is the number of single-match escapes, q is the host spacer acquisition probability, μ is the protospacer mutation probability, and β is the viral burst size. Here, the $n_{e}$ escapes are not necessarily unique. Note that, due to the low protospacer mutation probability μ , escape from host strains that share a double spacer match or higher is negligible. As the susceptible biomass S increases, the probability of at least one single-match escape approaches 1. The curves of ${\mathbb{P}}_{S}(n_{e}\geq 1)$ and expected number of escapes ${𝔼}_{S}(n_{e}\geq 1)$ in electronic supplementary material, figure S11, increase as a function of S for various parameters. The carrying capacity K , however, bounds the maximum achievable size of the susceptible host biomass S and, in effect, the number of viral variants generated. Naturally then, the probability of viral escape is limited by the intensity of host density-dependent competition. In other words, since max⁡S=K , we have that


(3.3) $$\max_{S}P_{S}\;(n_{e}\geq 1)=1-(q+(1-q)(1-\mu {)}^{\beta }{)}^{K}.$$

During SHC, competing host strains always partition the carrying capacity K which implies strictly $P_{S}\;(n_{e}\geq 1)<P_{K}(n_{e}\geq 1)$ .

**Figure 5.**
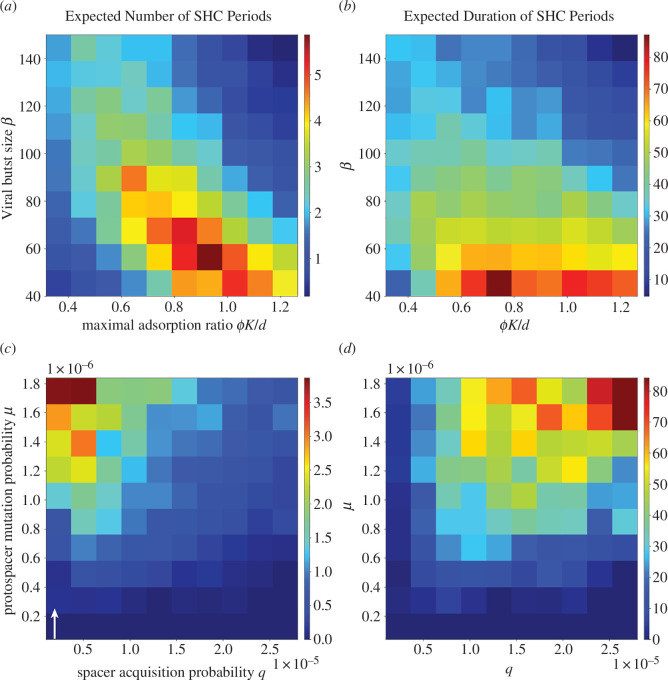
Diagrams of emergent trends in the alternating dynamics of the stochastic model of CRISPR-mediated microbe-lytic virus coevolution, as a function of dimensionless, composite parameters. In (*a,b*) the ecological parameters are varied, whereas in (*c,d*) the evolutionary ones are varied. Note that the colour bars assign colour values to the proportion of the 50 replicates that demonstrate a dynamical feature of interest. For (*a,b*), we fix the evolutionary parameters to be *protospacer mutation probability* $\mu =5\times 10^{-7}$ and *spacer acquisition probability* $q=10^{-5}$ , and for (*c,d*), we fix the ecological parameters to be *burst size* β=50 , and *maximal adsorption ratio* $\phi K/d=10^{-5}$ . (*a,b*) show the expected number and duration of SHC periods as ecological parameters are respectively varied. Note that zero SHC periods indicates no alternating dynamics. Alternating dynamics fail to emerge at significantly low and high β and ϕK/d . In the region of high β and ϕK/d , microbial extinction is commonplace and occurs rapidly, thus making SHC formation improbable. Below this region, the number of SHC increases and the duration shortens with the increase of β . (*b*,*c*) show the expected number and duration of SHC periods as evolutionary parameters are respectively varied. The number of SHC periods increases with μ and decreases with q . The duration of SHC periods increases with q and μ . As q is considerably increased, alternating dynamics fail to emerge due to rapid viral extinction (example simulations in electronic supplementary material, figure S5). At low q indicated by the white arrow, quasi-stationary dynamics corresponding to successive strain replacements are observed (see electronic supplementary material, figure S*5b*,*c,* e.g. realizations), akin to those observed in models of influenza (e.g. [14]).

Equation (3.3) establishes the relevant parameters for our sensitivity analysis. We consider the protospacer mutation probability upon infection μ , spacer acquisition probability q , viral burst size β and the carrying capacity K . To ensure that our parameters of interest are all non-dimensional, in place of K we consider the parameter that we refer to as the *maximal viral adsorption ratio* Kφ/d , representing the maximal number of host cells that can be adsorbed by the viral population within the lifetime of a virion. Dimensionless composite parameters reduce the number of parameters to a minimal set required to fully describe a system [20]. Furthermore, we develop an algorithm that identifies all candidate SHC periods, to identify the presence of alternating dynamics among simulations (detailed in electronic supplementary material, figure S8). We map the expected number and duration of SHC periods observed in a simulation (figure 5), and the mean times to total viral and host extinction (electronic supplementary material, figures S9 and S10).

We first consider our *ecological* parameters, viral burst size and maximal adsorption ratio, which relate to virus and host demography (parameter values listed in electronic supplementary material, table S1). Because we vary the maximal adsorption ratio via the carrying capacity, a high maximal adsorption ratio indicates weak density-dependent competition among the host population. Equation (3.3) indicates that increases in the burst size or maximal adsorption ratio should increase the probability of generating viral escapes and consequently accelerate the host succession process. In the extreme limits where burst size and maximal adsorption ratio are low, alternations rarely form as sufficient viral escapes are not generated, causing consequent rapid viral extinction. As burst size or maximal adsorption ratio increases, we observe that the expected number of SHC periods also increases (figure 5*a*,*b*). Duration of these SHC periods also shortens on average as burst size increases. Despite the shortening of durations, SHC periods re-emerge due to rare immune strains gaining the opportunity to establish a considerable abundance upon a transition to MVE. In general, as either burst size or maximal adsorption ratio increases, the probability of viral extinction decreases and that of host extinction increases (electronic supplementary material, figure S8*b*). At extremely large values of burst size and maximal adsorption ratio, SHC periods become significantly improbable, and the expected number and duration of SHC periods significantly decrease (electronic supplementary material, figure S8*b*). This suggests that the viral population generates a sufficient number of escapes to rapidly overcome the formation of SHC, and thus burns out the entire host population. In these extreme regions of phase space where large viral diversity is generated, we observe rapid host–virus oscillations that roughly resemble classic predator-prey limit cycles (e.g. electronic supplementary material, figure S5*d,e*).

Next, we consider our *evolutionary* parameters, spacer acquisition and protospacer mutation probability. In the extreme regimes of high spacer acquisition probability, an initial large viral epidemic is usually followed by rapid viral extinction, impeding the formation of alternations and thus SHC periods (see expected time to viral extinction in electronic supplementary material, figure S9*a*; electronic supplementary material, figure S6*a* depicts example simulation). The consequent high diversity of host immune strains limits both the number of susceptible hosts that the viral population can infect, and the host biomass that is available upon an escape from spacer matches. For a given spacer acquisition probability, the expected number and duration of respective SHC periods increase with protospacer mutation probability (figure 5*b*,*c*). This is also observed with increases in the maximal adsorption ratio (electronic supplementary material, figure S10*c*). For a fixed protospacer mutation probability (or the maximal adsorption ratio), decreasing the spacer acquisition probability causes SHC periods to shorten and re-emerge more frequently. Less immune diversity necessitates fewer escapes to transition to MVE, and in response rare immune strains can emerge. Moreover, we note that expected viral diversification upon an infection increases with viral repertoire size, analogously with protospacer mutation probability. In spite of this, under constant conditions of mutation and carrying capacity, an increase in repertoire size is not associated with an increasing trend of expected number and duration of SHC periods (electronic supplementary material, figure S10). This is a consequence of the single-match escape probability, and that of higher order ones, being independent of the repertoire size.

In addition to alternating dynamics, we identify a distinct non-equilibrium behaviour at relatively low spacer acquisition probabilities for a given protospacer mutation probability. These dynamics exhibit temporary periods of stationarity that intermittently transition in both viral and host immune strain composition; we refer to this as quasi-stationarity (examples in electronic supplementary material, figure S6*b*,*c*). Note that the total host population size does not necessarily reach carrying capacity. The quasi-stationarity region is indicated by a white arrow in figure 5*c*. At relatively lower protospacer mutation probabilities in the quasi-stationarity regime, one viral strain can dominate at a time, akin to patterns observed in phylodynamic models of influenza (e.g. [14]). As the protospacer mutation probability increases, periods of stationarity are more likely to establish with a coexistence of two or more viral strains. Despite the distinct phases, in general viral extinction is greater than zero before t=2000 , and the mean time to total viral extinction increases with protospacer mutation probability (electronic supplementary material, figure S9*a*).

## 4. Discussion

The alternating dynamics observed in a CRISPR-mediated microbe-lytic virus system is a recent addition to the repertoire of ‘selection dynamics’ established in the coevolutionary literature [4,5]. Here, we described how successive viral escapes and corresponding small outbreaks reorganize the strain structure of the host population, increasing its fragility and leading to a transition away from carrying capacity and a large lytic epidemic. This transition in population dynamics is accompanied by rapid host–pathogen co-diversification. We show that host density-dependent competition, along with heritable and adaptive immunity, is a key mechanism behind this transition. In its presence, small viral outbreaks increasingly destabilize host control by driving strain succession in the host and concentrating susceptible biomass in fewer escape pathways.

In host–pathogen models, classical directional, fluctuating and balancing selection have been primarily documented so far [8–11,13,14,21,22]. Punctuated selection, roughly resembling the virus-driven succession we study here, has been observed in an inverse gene-for-gene system [12]. Nevertheless, the successive rise of viral variants leading to explosive epidemics, and thus co-diversification, is unique to CRISPR-mediated coevolution. The CRISPR-mediated microbe-lytic virus system is perhaps the only modelled example of a host–pathogen system to date that exhibits a heritable adaptive immunity (sometimes referred to as a type of Lamarckian evolution; see [23,24]). In (inverse) matching allele and gene-for-gene models used for plant and microbial systems, pathogen resistance of hosts is not acquired, only inherited [8–12,21]. In strain-theoretic and phylodynamic models in human epidemiology, hosts acquire immunity throughout their lifetime but are typically born naive (i.e. there is no inheritance, see [13,14,22] for examples, although there can be brief and transient maternal immunity).

The adaptive immunity of host strains in our model has a conceptual analogy to habitat modification as implemented in a recent metapopulation model by Miller and Allesina [25]. Cyclical fluctuations and stable equilibria are, however, the only dynamics observed in that system. The contrasting dynamics of our model underscores that a comprehensive synthesis of the eco-evolutionary consequences of acquired memory, its inheritance and length remains to be developed. Understanding how eco-evolutionary mechanisms—e.g. density-dependent competition and heritable adaptive immunity—lead to different coevolutionary dynamics provides a foundation for such a synthesis. Furthermore, microbe-lytic virus systems fall under the class of predator-prey systems widely studied in ecology. Despite a variety of distinct fluctuating and equilibrium dynamics described for predator-prey coevolutionary models (e.g. [26–29]), to date, alternating dynamics have yet to be observed. Albeit, within a broader context, the alternating dynamics resemble critical behaviours observed in avalanche and seismological models [30,31]. Rigorously drawing this correspondence remains an avenue of future work.

Future studies of microbe-lytic virus coevolution should also consider variation of host phenotypes that exist alongside immune memory. This includes both variation in fixed fitness advantages and alternative lines of defence that confer specificity against natural enemies. A few theoretical studies have investigated the selection for constitutive and inducible defences in light of costs [32,33]—however, the role of intraspecific variation of frequency-dependent traits, conferred by defences, has yet to be established. Previous models and experimental work have made advances in correlating host competition to types of defence. Surface-modification-mediated resistance which confers constitutive costs has been shown to be selected in media with high resource availability, whereas CRISPR-Cas-mediated immunity which confers inducible costs is selected in media with low resource availability [34,35]. Our study shows that microbial extinction becomes increasingly likely at higher carrying capacities. This suggests that higher resource concentrations, which generate higher carrying capacities, can in turn promote microbial extinction. Namely, without explicitly considering costs due to viral exposure, instances of high resource availability could render CRISPR-Cas immunity ineffective due to the generation of excess viral diversity. These conditions would further strengthen selection for surface-modification-mediated resistance in microbial populations, as supported by the documented loss of the CRISPR-Cas system due to overwhelming generation of viral diversity via high protospacer mutation rates [36,37]. Furthermore, the observed effects of host carrying capacity also suggest that the presence of alternative negative density-dependent interactions, such as *interspecific* competition or predation, can aid in suppressing the genetic diversity of lytic viruses.

In the expected probability of evolutionary emergence $\left\langle P^{*}\right\rangle$ , we explicitly encoded the bipartite infection and tripartite escape network structures, and associated compositions of host abundance. This quantity complements other network-related metrics proposed in previous studies. In particular, Childs *et al*. have developed distributed immunity (DI) metrics which incorporate couplet and triplet motifs of the tripartite escape network [15], and have shown that high values of DI are associated with SHC regimes. In contrast to $\left\langle P^{*}\right\rangle$ , however, DI is not necessarily informative of the viral outbreaks/immune strain reorganizations that lead up to SHC-to-MVEs transitions. $\left\langle P^{*}\right\rangle$ includes susceptible host population size whereas DI does not. The total population size, as opposed to relative frequency, of the susceptible host provides direct insight into the number of replicates and escapes than be generated by a viral strain. Future work can improve upon the probability of emergence with a time-dependent analytical solution that explicitly accounts for the non-equilibrium dynamics of the host population.

Despite numerous theoretical studies of coexistence in microbe-virus systems [17,27,38,39], so far only a few have included strain-level variation and their emergent non-trivial interaction structures [4,5,40]. Our work here underscores the importance of considering strain-level variation and structure, and motivates further theoretical considerations of strain-level interactions that occur between microbial hosts and viral species with chronic and temperate ‘life histories’. Broader theoretical considerations of strain-level specificity, and consequent negative frequency-dependent selection, could aid in understanding dynamical patterns observed in competitive and syntrophic microbial systems (see [41] for long-term dynamical correlations that emerge with strain-level descriptions of microbial species).

Another direction for future work concerns the stabilization of the alternating population dynamics that emerge from CRISPR-induced coevolution. These dynamics are inherently transient due to the eventual extinction of the viral population. Interestingly, our model observations are supported by laboratory experiments that demonstrate rapid and repeatable phage extinctions when challenged with *Streptococcus thermophilus* [6]. Nevertheless, lytic viruses that elicit CRISPR-based defence, persist in natural settings. Future studies should therefore examine viable mechanisms that could enhance viral persistence. Model extensions that consider open populations and/or include space are of interest; so is the consideration of a larger network of ecological interactions. Alongside, further generation of field data on microbial CRISPR spacer arrays and viral genomes will aid in these theoretical developments by establishing network patterns that are relevant in a metacommunity context.

In the experimental context, dilution transfers of microbial cultures may cause the ‘washout’ of rare immune strains, which is analogous to extinctions due to drift (e.g. [42] for effects of different dilution factors in a competitive system). Our model has neglected host cellular losses by processes other than viral lysis, but conclusions are robust to the addition of a washout rate approximately an order of magnitude slower than the microbial replication rate. In particular, the alternating dynamics persist and the trends observed for the expected number and duration of the SHC periods remain similar to those without washout (electronic supplementary material, figure S11). A systematic exploration of the effects of host cellular losses on the emergence of alternating dynamics remains an area for future studies. Sufficiently large washout could reduce immune diversity during SHC, thus promoting transitions to MVEs. Extremely high washout rates could impede the construction of coevolution-generated immune structure, and in so doing, prevent the alternating dynamics altogether. Another source of stochastic losses is cellular death due to intrinsic causes such as senescence or other pathologies (e.g. developmental abnormalities or deleterious mutations). The prevalence of death due to intrinsic causes among microbes is poorly understood and associated measurements are extremely sparse, especially for those with CRISPR-Cas immunity. Future experimental work measuring cellular death rates will help further refine this demographic aspect of stochastic coevolutionary models.

The functioning of microbiomes greatly impacts the world around us: e.g. microbiomes of the human gut, those utilized for industrial and clinical processes, or those that mediate terrestrial and marine geochemistry [43–46]. The functioning of these microbiomes relies on the sustained abundances of constituent microbial populations, yet the occurrence of large viral epidemics can render any of the mentioned systems unstable. Our ability to understand and manage microbiomes hinges on considering how viral interactions affect microbial population dynamics and function. This in turn requires consideration of strain-level variation and structure.

## Data Availability

Code for model and analyses is publicly recorded on Zenodo [47] and hosted by a Github repository [48]. Analysis and model output used for figures are available on [49]. Supplementary material is available online.
